# Exploring the genomics of abiotic stress tolerance and crop resilience to climate change

**DOI:** 10.1002/tpg2.20445

**Published:** 2024-03-13

**Authors:** Rajeev K. Varshney, Rutwik Barmukh, Alison Bentley, Henry T. Nguyen

**Affiliations:** ^1^ WA State Agricultural Biotechnology Centre, Centre for Crop and Food Innovation Food Futures Institute, Murdoch University Murdoch Western Australia Australia; ^2^ ANU College of Science The Australian National University Canberra Australian Capital Territory Australia; ^3^ Division of Plant Science and Technology University of Missouri Columbia Missouri USA

Abiotic stresses have a detrimental impact on crop production globally. The escalating frequency and intensity of these stresses, driven by rapid changes in climatic conditions, pose significant challenges to agriculture. This situation is worsened by the burgeoning human population that is projected to heighten the demand for food in the coming years, emphasizing the need for further agricultural innovations. Addressing these challenges and steering agriculture toward sustainability requires concerted research efforts to mitigate the adverse effects of climate change‐induced abiotic stresses. One of the most logical and cost‐effective strategies on a global scale is the development and utilization of crop varieties endowed with increased tolerance to different abiotic stresses.

Conventional breeding has played a key role in developing crops resilient to abiotic stress; however, recent advancements in genomics technologies have expedited these efforts. The development and public availability of multiple crop genomes, coupled with improvements in genome assembly quality and the emergence of pangenome and super‐pangenome, signify a substantial leap forward. High‐quality reference genomes, whole‐genome resequencing, and pangenome approaches have enabled the mapping of allelic variants, discovery of candidate genes, development of molecular markers, and the introgression of traits related to abiotic stress tolerance. This wealth of genomic data, complemented by other omics datasets such as transcriptomics, proteomics, metabolomics, and phenomics, has effectively bridged the genotype–phenotype gap (Varshney, Bohra et al., 2021). This integration is crucial for gene mapping and marker‐assisted breeding, offering a comprehensive understanding of crop responses to diverse abiotic stresses at the whole‐genome level. Identifying and characterizing candidate genes and elucidating biological mechanisms underpinning abiotic stress tolerance using modern genomic technologies are essential for designing climate‐resilient crops for the future.

The current special section titled “Genomics of abiotic stress tolerance and crop resilience to climate change” comprises 16 research articles, five reviews, and one perspective. These articles cover diverse topics such as genome‐wide association studies (GWAS), long noncoding RNA transcriptome profiling, proteome analysis, integrated multi‐omics analysis, genome‐wide identification of key abiotic stress tolerance genes, abiotic stress tolerance mechanisms, and the application of pangenomics and machine learning in identifying genes/proteins associated with abiotic stress responses. Collectively, these articles underscore the potential of genomic innovations in providing fresh insights into plant responses and tolerance to various abiotic stresses, comprising drought, temperature extremes, salinity, anaerobic germination, and pre‐harvest sprouting (PHS), across various crop species.

## 1 DROUGHT TOLERANCE

Over time, crops have developed diverse morphological, physiological, and molecular mechanisms to counteract the effects of drought stress (Barmukh et al., 2022; Vadez et al., 2024). While most breeding efforts to improve drought tolerance have concentrated on above‐ground traits, focusing on high yields under optimal environments, there has been relatively limited emphasis on below‐ground traits. Root system architecture emerges as a prominent target for breeding crops that can proficiently use available resources and thrive under drought stress (Varshney, Barmukh, et al., 2021). In this special section, Kalra et al. (2023) outlined recent advancements in comprehending the role of roots and root‐mediated plant responses in enhancing crop survival and productivity under drought conditions. This article provided a summary of diverse genes, transcription factors, and quantitative trait loci (QTLs) involved in governing root growth and development during drought. Additionally, it discussed various root phenotyping methods applicable for understanding alterations in root system architecture in field conditions.

Given the complex nature of drought as a trait, both linkage‐ and association‐mapping analyses have been undertaken to uncover the genetic mechanisms underpinning drought tolerance (Kushwah et al., 2022; Xiong et al., 2023). In recent years, specialized mapping populations involving the crossing of multiple parental lines, for example, the multi‐parent advanced generation intercross (MAGIC) (Cavanagh et al., 2008), have been employed to genetically dissect complex traits. In this special section, Thudi et al. (2023) utilized the desi chickpea MAGIC population for fine mapping of drought tolerance in chickpeas. GWAS identified 737 markers significantly linked to crucial agronomic traits, including days to 50% flowering, days to maturity, biomass, and 100‐seed weight, among others. The study identified key candidate genes like *CaTIFY4b* and *FRIGIDA* that hold potential for enhancing drought tolerance in chickpea. In another study, Ferrão et al. (2023) integrated GWAS and diallel analyses to investigate the genetic basis of key agronomic traits evaluated under drought environments in coffee. Molecular markers with major effects associated with disease resistance and post‐harvest traits were identified, while yield and plant architecture displayed a polygenic background. This study underscored the significance of nonadditive gene actions and highlighted hybrid vigor when genotypes from different geographically botanical groups are crossed, paving the way for implementing molecular breeding to expedite coffee improvement.

Nitrogen is essential for plant growth and productivity, and understanding the mechanistic basis of nitrogen use efficiency (NUE) under drought conditions is crucial for improving crop yield. Through GWAS analysis, Koua et al. (2023) identified 27 QTLs strongly linked to drought‐related traits and 10 QTLs associated with NUE traits in wheat. Chromosomes 1B and 5A were found to harbor pertinent genomic loci containing potential genes such as amino acid transporters and cold shock proteins, with pleiotropic effects on enhanced NUE under drought stress.

Apart from genomics, diverse single‐ and multi‐omics strategies have been used to unravel the molecular foundations of drought tolerance in crops. For instance, long noncoding RNAs (lncRNAs) serve as crucial regulators of transcription and gene expressions at the post‐transcriptional level, influencing RNA stability and translation (Chen et al., 2016). In mulberry, Ackah et al. (2022) unearthed novel lncRNAs and protein‐coding mRNAs, elucidating the intricate network of interactions between lncRNAs, protein‐coding mRNAs, and their miRNA precursors in response to drought stress. This investigation demonstrated that target genes of differentially expressed lncRNAs were notably enriched in the biosynthesis of secondary metabolites, providing fresh insights into mulberry‐drought stress interactions. Furthermore, proteomics stands out as a potent omics technique capable of shedding light on the complex molecular mechanisms linked with drought tolerance by analyzing the expression profile of the proteome. Nouraei et al. (2023) utilized isobaric tags for relative and absolute quantitation proteomic analysis on near‐isogenic lines, identifying five key proteins underlying the *qDSI.4B.1* QTL on the short arm of chromosome 4B conferring drought tolerance in wheat. Additionally, in their investigation of the molecular basis of drought stress response in chickpea, Kudapa et al. (2023) adopted an integrative “multi‐omics” approach, demonstrating the differential accumulation of transcripts, proteins, and metabolites in root tissues under drought conditions. The integration of transcriptomics and proteomics data unveiled hub proteins, encoding isoflavone 4′‐O‐methyltransferase and UDP‐d‐glucose/UDP‐d‐galactose 4‐epimerase, involved in pathways such as antibiotic biosynthesis, galactose metabolism, and isoflavonoid biosynthesis to activate drought stress response mechanisms. Moreover, the integration of metabolomics data identified six metabolites exhibiting a significant correlation with galactose metabolism under drought stress.

## 2 ANAEROBIC GERMINATION TOLERANCE

One of the mechanisms that enable crops like rice to tolerate germination under submerged conditions involves the rapid degradation of starch reserves in seeds during hypoxic/anoxic conditions, facilitating the swift growth of the coleoptile to escape the stress. Although earlier research has primarily delved into the physiological aspects of oxygen stress, only few investigations have explored the broad spectrum of natural variation in traits related to anaerobic germination tolerance in rice. Thapa et al. (2022) conducted a GWAS analysis on a rice diversity panel comprising 241 accessions, revealing 30 significant marker‐trait associations (MTAs). Among these MTAs, 14 colocalized with previously detected genes linked to anaerobic germination tolerance, while 16 were novel discoveries. Furthermore, the integration of transcriptome analysis with GWAS identified 77 potential genes controlling anaerobic germination tolerance.

## 3 EXTREME TEMPERATURES: HEAT AND COLD STRESS TOLERANCE

The pace of plant growth and development is predominantly influenced by the ambient temperature prevailing during the plant's cultivation. With the rising fluctuations in environmental conditions, extreme temperature events are anticipated to intensify in frequency and duration. Heat stress induced by climate change is detrimentally affecting global crop production, disrupting biochemical, physiological, and metabolic processes, and consequently impeding growth, development, and yield (Sato et al., 2024). Maize is vulnerable to heat stress, particularly during the flowering and early grain‐filling stages. Within this special section, Djalovic et al. (2023) succinctly summarize recent advancements in comprehending the repercussions of heat stress on maize. The discussion underscored the implication of heat stress‐related trait phenotyping and elucidated key molecular mechanisms that aid in identifying novel sources for heat stress tolerance, offering opportunities to enhance heat tolerance in maize. Furthermore, in the exploration of terminal heat tolerance mechanisms in wheat, Sihag et al. (2023) conducted biochemical and gene expression analyses using two heat‐tolerant and two heat‐sensitive genotypes. The analysis revealed a notable increase in total soluble sugar, proline, and glycine betaine contents in the flag leaf, alongside a reduction in grain‐filling duration, 1000‐kernel weight, and grain yield under heat stress. Additionally, the expression of heat‐responsive genes linked to heat shock proteins, β‐glucan synthesis, and flavonoid biosynthesis was observed to be elevated in heat‐tolerant wheat genotypes compared to their heat‐sensitive counterparts.

Low temperature represents another damaging abiotic stress that is on the rise in frequency due to climate change. Cold stress stimulates cellular changes, causing oxidative stress, a slowdown in metabolism, growth limitations, and ultimately a decline in crop productivity (Soualihou et al., 2022). While extensive knowledge on the influence of cold stress on perception, signal transduction, gene expression, and metabolism are readily available in *Arabidopsis thaliana*, there is a relative scarcity of such information in major crops. Jan et al. (2023) discuss, in this special section, the effect of cold stress on the growth of staple food crops. They emphasized the mechanism of cold perception and delved into the role of various sensors and transducers in cold signaling. The review encapsulated advancements in cold tolerance research at the genome, transcriptome, proteome, and metabolome levels, highlighting how these insights present opportunities for designing cold‐tolerant crops.

## 4 SOIL CONSTRAINTS AND SALINITY TOLERANCE

Plant roots frequently encounter unfavorable soil conditions that impose limitations on plant growth and yield. Soil constraints, including acidity, salinity, sodicity, and dispersion, constitute significant contributors to crop yield losses, particularly in the dryland regions. In response to dynamic environments, plants use adaptive strategies such as phenotypic plasticity to facilitate shifts in phenotypes. Within this special section, Bhoite et al. (2022) presented an overview of advantageous alleles underpinning enhanced gene expression, QTLs, and epigenetic regulation governing plant responses to soil constraints. They outlined a strategy for exploring quantitative traits in wheat, involving the investigation of significant alleles, functional characterization of variants, gene validation using advanced genomic tools, and marker development for molecular breeding and genome editing. Additionally, the advancements in gene editing in wheat were underscored to control plasticity traits in constrained soil conditions.

Elevated concentrations of NaCl in the soil lead to a decrease in water potential and diminished water availability, resulting in osmotic stress in plants (Acosta‐Motos et al., 2017). Acharya et al. (2023) conducted genome‐wide identifications and phylogenetic analyses of key genes, including *NHX*, *CIPK*, and *CBL*, associated with the plant salt overly sensitive (SOS) signaling pathway across six *Rosaceae* species. The investigation revealed that intron‐rich *CIPKs* in *Rosaceae* and *Arabidopsis* traced their origins back to algae *CIPKs* and those found in early plants, suggesting the evolutionary development of intron‐less *CIPKs* from their intron‐rich counterparts. The findings indicated a high degree of conservation in the SOS signaling pathway in *Prunus persica*. Furthermore, an integrated multi‐omics analysis, followed by targeted metabolomics, hormone, and ion analyses of date palm roots and leaves, unveiled the effective integration of diverse salt‐tolerance mechanisms observed in both halophytes and glycophytes (Mueller et al., 2023). The date palm demonstrated “avoidance” through proficient exclusion of sodium and chloride ions at the roots, coupled with “acclimation” mechanisms such as osmotic adjustment, scavenging of reactive oxygen species in leaves, and restructuring of the ribosome‐associated proteome in root cells exposed to salt.

## 5 PRE‐HARVEST SPROUTING RESISTANCE

The premature germination of seeds, referred to as PHS, poses a significant challenge in world‐wide wheat production, causing a decline in the bread‐making quality of affected grain. Breeding for resistance to PHS is crucial to mitigate losses in suboptimal conditions. Despite the existence of a wheat reference and pangenome, a comprehensive understanding of the mechanisms governing different sources of PHS resistance remains limited. In this special section, Dallinger et al. (2023) conducted a GWAS analysis and identified compelling evidence for novel QTLs associated with PHS resistance on chromosomes 1A and 5B. Several peaks on chromosome 4A were found to co‐localize with the prominent resistance locus *Phs‐A1*, containing causal genes *TaPM19* and *TaMKK3*. The mapping of markers and genes to the pangenome and chromosomal alignments provided insights into the structural variations underlying this key PHS‐resistance locus.

## 6 DISCOVERY OF CANDIDATE GENES ASSOCIATED WITH STRESS RESPONSES

Examining a set of gene families can provide major insights into their specific or overlapping functions, aiding in the detection of candidate gene(s) participating in vital biological processes, including responses to abiotic stress. The R2R3‐MYB transcription factor family controls plant development and stress resistance, and the family's gene count varies significantly across different plant types. In this special section, Yao et al. (2022) conducted a comprehensive genome‐wide analysis of R2R3‐MYB genes in ginger, uncovering 299 potential *ZoMYB* genes categorized into eight groups. The analysis of gene duplication revealed 120 instances of segmental duplications in the ginger genome. Examining the gene expression patterns of 10 *ZoMYBs* in ginger leaves subjected to abscisic acid and low‐temperature stress treatments unveiled potential roles of *ZoMYBs* in anti‐stress responses in ginger.

## 7 GENETIC DIVERSITY AND STRESS TOLERANCE

To address the impending global food demand, it is imperative to elevate genetic gain in the breeding program by incorporating a broader spectrum of genetic diversity to enhance crop tolerance to environmental stressors induced by climate change (Sinha et al., 2021). Introgression lines, established through several generations of crossing and/or backcrossing with the aid of marker‐assisted selection, serve as a stable genetic resource. Horsnell et al. (2023) detailed the development of chromosome segment substitution lines in spring wheat, aiming to comprehensively capture genetic variation from tetraploid (*Triticum turgidum* ssp. *dicoccoides*) and diploid (*Aegilops tauschii*) progenitor species. The characterization of this population at both genotypic and phenotypic levels showcased the potential utility of these lines for pre‐breeding and breeding research to advance stress tolerance in wheat.

## 8 GENOMICS AND MACHINE LEARNING ADVANCES FOR DEVELOPING CLIMATE‐RESILIENT CROPS

In recent years, the rapid progress in sequencing technologies has resulted in a transformative impact on plant science research. Technologies like restriction‐site‐associated DNA sequencing (RADseq/GBS) have opened avenues for acquiring precise information on population genetic diversity, significantly expediting the exploration of adaptive evolutionary mechanisms (Feng et al., 2020). Dang et al. (2023) employed RADseq technology for a population genomic analysis, unveiling the genetic variation and structure of *Reaumuria trigyna*. The analysis identified probable sites under diversifying selection in genes associated with phytohormone regulation and the synthesis of secondary metabolites, providing major insights into the adaptive diversification of *R. trigyna* species.

Furthermore, many genes absent in reference accessions possess functions of potential adaptive significance, making the creation of a pan‐genome a crucial task for globally important crops. To identify the genomic diversity existing within cowpea domesticates, Liang et al. (2023) generated de novo genome assemblies and constructed a pangenome containing 26,026 core, 4963 noncore, and 35,436 pan genes. Among the noncore genes, gene ontology terms related to stress and defense response were highly enriched among noncore genes, whereas core genes exhibited enrichment in terms linked to transcription factor activity and metabolic processes. Noncore genes harbored a higher ratio of potentially disruptive modifications than core genes, suggesting a significant contribution of noncore genes to the diversity existing within domesticated species of cowpea.

The identification of numerous genes responsive to abiotic stress is necessary for breeding crops that exhibit tolerance to such stressors. Machine learning algorithms, involving support vector machines, random forests, and adaptive boosting, offer the promise to predict stress‐responsive genes with increased accuracy. Meher et al. (2022) crafted a computational model called ASRpro, employing machine learning algorithms to identify genes responsive to six distinct abiotic stresses in plants. This proposed model has the capability to complement transcriptome profiling, comparative genomics, and GWAS for discovering genes controlling abiotic stress in crop plants.

## 9 FUTURE PERSPECTIVES

Modest projections of a 2°C increase in the global mean temperature suggest forthcoming alterations in the timing and severity of abiotic stresses. To prevent further environmental damage, it is imperative to boost the productivity of staple crops without expanding agricultural land area and by curbing the usage of water and fertilizers. Considering this, Robles‐Zazueta et al. (2023) advocated for the combination of abundant data from crop breeding programs with increasingly cost‐effective omics tools to predict wheat performance under diverse climate change scenarios. Additionally, breeders can strategically formulate and deliver future wheat ideotypes by leveraging an enhanced comprehension of the genetic and physiological processes activated when the crop faces combinations of stresses.

In the coming years, crop breeding necessitates a shift toward more participatory approaches that align with evolving market demands. This transition should concurrently promote the strategic deployment of novel traits for the sustainable intensification of agriculture while revitalizing the cultivar development pipeline to achieve higher rates of genetic gains in farmers’ fields. Bassi et al. (2023) offered insights into how plant breeding efforts can either boost or diminish genetic gains based on the breeder's equation. Given the vast array of natural variations for tolerance to abiotic stresses within cultivated and wild germplasm resources, future research should concentrate on mining key genes and their superior alleles related to abiotic stress. Moreover, gene pyramiding can be explored as a strategy to develop crop varieties adapted to multiple stresses. The articles featured in this special section not only contribute to enriching our knowledge of the molecular basis of plants’ adaptive responses to abiotic stresses but also hold potential for successful breeding of crop varieties adapted to climate change using marker‐assisted selection and genome editing tools.

## AUTHOR CONTRIBUTIONS

**Rajeev K. Varshney**: Writing—original draft; writing—review and editing. **Rutwik Barmukh**: Writing—review and editing. **Alison Bentley**: Writing—review and editing. **Henry T. Nguyen**: Writing—review and editing.
